# Evaluation of Sub cutaneousRush Immunotherapy Effectiveness in Perennial Allergic Rhinitis after a Year from Treatment

**Published:** 2019-05

**Authors:** Mohsen Tizro, Reza Farid Hosseini, Maryam Khoshkhui, Ali Fouladvand, Mojgan Mohammadi, Samane Sistani, Farahzad Jabbari Azad

**Affiliations:** 1 *Department of Pediatric , Faculty of Medicine, Zahedan University of Medical Sciences Zahedan, Iran.*; 2 *Allergy Research Center, Mashhad University of Medical Sciences, Mashhad, Iran. *; 3 *Department of Pediatric , Shahid Rahimi Hospital, Lorestan University of Medical Sciences, Khorram Abad, Iran.*; 4 *Department of Biomedical Informatics, Facultyof Medicine, Mashhad University of Medical Sciences, Mashhad, Iran.*

**Keywords:** Allergic rhinitis, Immunotherapy, Rush immunotherapy

## Abstract

**Introduction:**

Allergen immunotherapy is an effective treatment for allergic rhinitis. Conventional immunotherapy takes at least 5 to 6 months to reach the maintenance dosage; nonetheless, rush immunotherapy accelerates to reach the maintenance dose several months earlier. However, the safety and efficacy of this treatment has not been widely investigated. The objective of the present study was to determine the efficacy of subcutaneous rush immunotherapy in the patients with perennial allergic rhinitis after a year from treatment.

**Materials and Methods:**

This study was carried out on a total of 15 patients with allergic rhinitis who received rush immunotherapy and were evaluated for the quality of life and clinical symptoms improvement with Sino-Nasal Outcome Test Questionnaire (SNOT-22) and Mini Rhino conjunctivitis Quality of Life Questionnaire (RQLQ) before and after a year from treatment. Moreover, specific weed mix Immunoglobulin E (IgE) was measured before and after a year from treatment. Statistical analysis was performed using SPSS software (version 16) (P<0.05).

**Results:**

The comparison of specific IgE indicated a significant reduction between before and after a year from treatment (P=0.005for pigweed)(P=0.022 for salsola). There was a significant decrease in clinical symptoms according to SNOT-22 Questionnaire [(mean score: 46.00, before the treatment) and (mean score: 14.06, after the treatment)]. The quality of life for most of the patients was moderate (46.7%) before the treatment and good (80%) after the treatment, which was considered statistically significant (P>0.001).

**Conclusion:**

Rush immunotherapy is an effective treatment in the patients with allergic rhinitis. It seems to be an alternative treatment in cases that need more rapid treatment. However, it is recommended to carry out other studies on the control group.

## Introduction

Allergic rhinitis involves nasal congestion, rhinorrhea (usually thin and clear), sneezing, itchy nose, itchy palate, itchy throat, ear or face (1). Since the end of the past century, there has been a rise in the incidence of allergic diseases and specifically rhinitis (2). Aeroallergens are the important causes of allergic rhinitis. Weed allergens, especially salsola, are the most common aeroallergens involved in allergic diseases in the north region of Iran (3). Specific immunotherapy was introduced by Noon and Freeman in 1911 to treat allergic rhinitis (4,5). Different studies have confirmed the effectiveness and immunity of allergen immunotherapy in seasonal or permanent allergic rhinitis (6-8). 

Initially, immunotherapy leads to an increase in specific Immunoglobulin E (IgE). Then, there is a gradual but progressive reduction in IgE levels towards the initial or even lower levels (9). Subcutaneous immunotherapy can be simple, rush, ultra-rush or cluster (10). The common method of subcutaneous immunotherapy is weekly injections that are time-consuming and take 7 to 8 months to reach the maintenance dose of injection. Therefore, the treatment would show to be effective later. It also involves high costs that make some patients reluctant to continue the full treatment.

Rush and ultra-rush immunotherapies have the advantage of reaching the maintenance dose and clinical improvement. They also help reduce the frequency of visits and overall cost (5). Systematic reactions to premedication managed to reach 25% from 73% with oral antihistamines and corticosteroids (9). Rush and ultra-rush immunotherapies have many advantages over ordinary immunotherapy programs in selective patients. These advantages include 1) reduced time of reaching the maintenance dose, 2) probable rapid reduction of allergen-specific IgE, an increase in allergen-specific Immunoglobulin G4, increase in interleukin 10, transforming growth factor beta and Foxp3 induction, as well as 3) economical use due to the reduced number of injections and visits (11). 

Therefore, in the present clinical trial, the effectiveness of accelerated subcutaneous rush immunotherapy was evaluated on the patients afflicted with permanent allergic rhinitis after a year from treatment. All the reported rush immunotherapy protocols were multi-dose, one-day, along with a weekly gradual increasing dose plan in order to reach a monthly maintenance dose that took about 8 to 11 weeks. Consequently, the decision was made to develop a corrected protocol with a three-day accelerated rush immunotherapy to reach the monthly maintenance dose. In practice, the weekly build-up program was omitted and the patients reached the maintenance dose on the third day.

## Materials and Methods

In this study, 15 patients participated with permanent allergic rhinitis and allergic to salsola or pigweed diagnosed using a prick test. The subjects were within the age range of 15 to 55 years old and were treated with a three-day accelerated rush immunotherapy protocol, as well as the weed mix extract (Greer Co., US). The present study (Code: 940678) was authorized by the ethics committee of Mashhad University of Medical Sciences and the written consent was obtained from all the patients for the participation and treatment in the Allergy and Immunology section of Ghaem hospital in Mashhad. 

This study started from June 2015 and lasted up to October 2016. The blood samples of 5 cc were taken from the brachial regions of the subjects before the accelerated rush immunotherapy. The samples were centrifuged and the resultant serum was examined for specific IgE against weed mix using enzyme-linked immunosorbent assay (ELISA) and preserved at -80°C. Moreover, before the initiation of the immunotherapy, the intensity of clinical symptoms was checked in Sino-Nasal Outcome Test Questionnaire (SNOT-22) and Mini Rhino conjunctivitis Quality of Life Questionnaire (RQLQ).

In this immunotherapy method, before the beginning of protocol and according to it, the patients were given prednisolone (30 mg) every 12 h, ranitidine (150 mg) every 12 h, Airokast or Montelukast (10 mg) every day, Telfast or Fexofenadine (180 mg) every 12 h. Once a patient showed a systematic reaction, the protocol would stop. However, in later visits, an increasing dose of 0.05 ml was added to the last dose to finally reach the maintenance dose. Out of 21 patients who entered the study, 15 cases managed to continue the protocol.

The standardized SNOT-22 questionnaires were completed once again after a year from the beginning of immunotherapy with a maintenance dose. Then this information was compared with that collected before the protocol. The blood samples were taken from all the subjects to examine the specific IgE against weed mix. These samples were preserved in -80°C. All pre- and post-immunotherapy samples were supposed to be examined by means of Elisa kit. The IgE was investigated again after a year and compared with that of the pre-treatment period. To study the specific IgE of weed mix aeroallergen in the laboratory, the two salsola and pigweed kits were used (ASTRA BIOTECH, Germany). All statistical analyses were carried out by SPSS software (version 16) and P-value was considered statistically significant (P˂0.05). 

## Results

Table 1 tabulates the demographic information and concomitant diseases with allergic rhinitis in the studied subjects. Out of 15 participants, 8 (53.3%) and 7(46.7%) cases were female and male, respectively. The subjects were within the age range of 35.07 years. Moreover, 33.30% of the patients with allergic rhinitis suffered from food allergy. In addition, 6.67% of the cases suffered from asthma besides allergic rhinitis.

**Table 1:** Demographic information and concomitant diseases with allergic rhinitis

	**Demographic ** **information**	**Finding**
	Total Number of patients	15
	Age (Mean±Standard deviation)	35.07
Gender: Number (%) of patients	Male	7 (46.70%)
	Female	8 (53.30%)
Diagnosis: Number (%) of patients	Allergic rhinitis	9 (60%)
	Allergic rhinitis + Asthma	1 (6.67%)
	Allergic rhinitis + Food Allergy	5 (33.30%)

The mean scores obtained from SNOT-22 questionnaire were 46 and 14.06before and after the immunotherapy, respectively, with the significance level of P=0.002. Patients’ nasal and optical symptoms, daily activities, and irritating problems were rated on the Mini RQLQ and then separately summed up. The mean scores of the symptoms before and after a year from treatment were compared and showed a statistically significant reduction (P˂0.05). The mean scores of the serum level of specific pigweed IgE were estimated as 5.37 and 0.47 before and after a year from treatment, respectively. These were compared through the Wilcoxon signed-rank test and the results demonstrated a significant decrease (P=0.05). 

**Fig 1:**
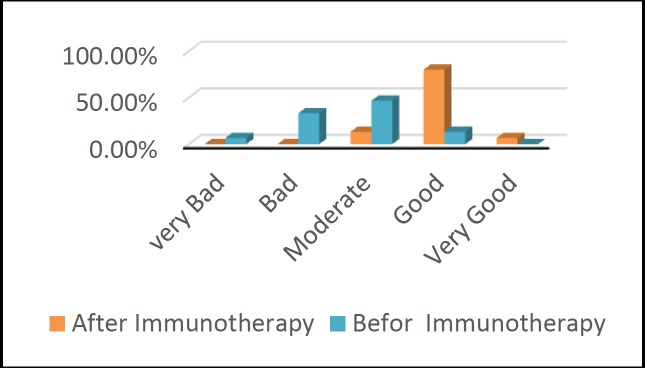
Health self-assessment

The mean scores of the serum level of specific salsola IgE were calculated as 35.79 and 2.82before and after a year from treatment, respectively. The obtained scores were compared using the Wilcoxon signed-rank test showing a significant reduction (P=0.022). Self-rating scores of patients’ health with permanent allergic rhinitis were compared before and after the accelerated rush immunotherapy. The health state of the majority of subjects (46.7%) before the treatment was moderate. After the immunotherapy, the health state of the majority of patients (80%) was categorized as good. This improvement in health indicated a significant difference (P˂0.001) (Fig.1).

## Discussion

In simple immunotherapy, in order to reach the therapeutic effect, there is a need for at least 5 to 6 months of build-up. In the accelerated rush immunotherapy, within three days of hospitalization, a patient would reach the maintenance dose. Therefore, by employing this schedule, the number of visits, duration of immunotherapy period, and overall treatment costs would be decreased. To reduce and control the costs of systematic side effects, the premedication was carried out three days before the protocol and during the prescribed protocol. In addition, the immunotherapy was performed in the hospital.

Schwante et al. investigated the patients’ quality of life after subcutaneous and sublingual immunotherapies through the completion of Mini RQLQ by subjects after six months and a year from treatment. The results revealed higher quality of life in both groups (12). Moreover, Filanowicz et al. analyzed the life quality of patients with asthma and allergic rhinitis after immunotherapy through the completion of Mini RQLQ and SNOT-22 questionnaire. According to the findings, it was realized that immunotherapy managed to raise the quality of life to a significant extent in this group of patients (13). 

Klunker et al. applied a rush-immunotherapy with ragweed, along with a premedication of Omalizumab. Roger et al. evaluated the quality of life and occupational and academic preparation of allergic rhinitis patients after allergen immunotherapy. In the aforementioned study, a strong effect was observed on academic and occupational preparation, as well as the quality of life of the studied patients. All the above-mentioned results are in line with findings of the present study. 

In a study carried out by Angele et al. on 217 allergic rhinitis patients in 2017, the specific IgE level was measured and its correlation with the severity of allergic rhinitis was investigated. Based on the results, it was revealed that the specific IgE level was a reliable biomarker of the intensity of allergic rhinitis symptoms (14). This result is also consistent with the findings of the present study. The limitations of the present study were the number of participants and patients' dissatisfaction for the admission to the hospital.

## Conclusion

Rush Immunotherapy seems to be effective in the improvement of the clinical symptoms and reduction of the serum level of allergen-specific IgE. In cases with the purpose of reaching a faster response level or when the matter of distance might disrupt the reception of immunotherapy doses, the accelerated rush immunotherapy protocol can replace simple immunotherapy. 

## References

[1] Di Lorenzo G, Pacor ML, Amodio E, Leto-Barone MS, La Piana S, D’Alcamo A (2011). Differences and similarities between allergic and nonallergic rhinitis in a large sample of adult patients with rhinitis symptoms. International archives of allergy and immunology.

[2] Zhang L, Han D, Huang D, Wu Y, Dong Z, Xu G (2009). Prevalence of self-reported allergic rhinitis in eleven major cities in china. International archives of allergy and immunology.

[3] Mahboubi Oskouei Y, Farid Hosseini R, Ahanchian H, Jarahi L, Ariaee N, Jabbari Azad F (2017). Report of Common Aeroallergens among Allergic Patients in Northeastern Iran. Iranian journal of Otorhinolaryngology.

[4] Dursun A, Sin B, Oner F, Misirligil Z (2006). The safety of allergen immunotherapy (IT) in Turkey. Journal of investigational allergology and clinical immunology.

[5] Brüggenjürgen B, Reinhold T, Brehler R, Laake E, Wiese G, Machate U (2008). Cost-effectiveness of specific subcutaneous immunotherapy in patients with allergic rhinitis and allergic asthma. Annals of Allergy, Asthma&Immunology.

[6] Walker S, Varney V, Gaga M, Jacobson M, Durham S (1995). Grass pollen immunotherapy: efficacy and safety during a 4‐year follow‐up study. Allergy.

[7] Cox L, Calderon MA (2010). Subcutaneous specific immunotherapy for seasonal allergic rhinitis: a review of treatment practicesin the US and Europe. Current medical research and opinion.

[8] Wambre E, DeLong JH, James EA, LaFond RE, Robinson D, Kwok WW (2012). Differentiation stage determines pathologic and protective allergen-specific CD4+ T-cell outcomes during specific immunotherapy. Journal of Allergy and Clinical Immunology.

[9] Moote W, Kim H (2011). Allergen-specific immunotherapy. Allergy, Asthma & Clinical Immunology.

[10] Sheikh A, Hurwitz B, Shehata Y (2007). House dust mite avoidance measures for perennial allergic rhinitis. Cochrane Database Syst Rev.

[11] SchwankeT, Carragee E, Bremberg M, Reisacher WR (2017). Quality-of-life outcomes in patients who underwent subcutaneous immunotherapy and sublingual immunotherapy in a real-world clinical setting. American journal of rhinology & allergy.

[12] Roger A, Campillo EA, Torres M, Millan C, Jáuregui I, Mohedano E (2016). Reduced work/academic performance and quality of life in patients with allergic rhinitis and impact of allergen immunotherapy. Allergy, Asthma & Clinical Immunology.

[13] Corsico AG, De Amici M, Ronzoni V, Giunta V, Mennitti MC, Viscardi A (2017). Allergen-specific immunoglobulin E and allergic rhinitis severity. Allergy & Rhinology.

[14] Lee J-H, Kim S-H, Lee WY, Yong SJ, Shin KC, Lee MK (2013). Immunotherapy -2079 Comparisonof house dust mite specific IgE and IgG4 between patients receiving rush immunotheraopy and conventional immunotherapy. World Allergy Organization Journal.

